# Anticancer osmium complex inhibitors of the HIF-1α and p300 protein-protein interaction

**DOI:** 10.1038/srep42860

**Published:** 2017-02-22

**Authors:** Chao Yang, Wanhe Wang, Guo-Dong Li, Hai-Jing Zhong, Zhen-Zhen Dong, Chun-Yuen Wong, Daniel W. J. Kwong, Dik-Lung Ma, Chung-Hang Leung

**Affiliations:** 1 State Key Laboratory of Quality Research in Chinese Medicine, Institute of Chinese Medical Sciences, University of Macau, Macao, China; 2 Department of Chemistry, Hong Kong Baptist University, Kowloon Tong, Hong Kong, China; 3 Department of Biology and Chemistry, City University of Hong Kong, Tat Chee Avenue, Kowloon, Hong Kong SAR, China

## Abstract

The hypoxia inducible factor (HIF) pathway has been considered to be an attractive anti-cancer target. One strategy to inhibit HIF activity is through the disruption of the HIF-1α–p300 protein-protein interaction. We report herein the identification of an osmium(II) complex as the first metal-based inhibitor of the HIF-1α–p300 interaction. We evaluated the effect of complex **1** on HIF-1α signaling pathway *in vitro* and *in cellulo* by using the dual luciferase reporter assay, co-immunoprecipitation assay, and immunoblot assay. Complex **1** exhibited a dose-dependent inhibition of HRE-driven luciferase activity, with an IC_50_ value of 1.22 μM. Complex **1** interfered with the HIF-1α–p300 interaction as revealed by a dose-dependent reduction of p300 co-precipitated with HIF-1α as the concentration of complex **1** was increased. Complex **1** repressed the phosphorylation of SRC, AKT and STAT3, and had no discernible effect on the activity of NF-κB. We anticipate that complex **1** could be utilized as a promising scaffold for the further development of more potent HIF-1α inhibitors for anti-cancer treatment.

Transition metal complexes have attracted increasing interest in recent years for a variety of different applications, including luminescent sensing, as catalysts for DNA and RNA cleavage reactions, or single-molecule magnets^1^. In medicinal chemistry, metal complexes have emerged as viable scaffolds for the development of therapeutic agents targeting proteins or DNA^2^. Structurally, transition metal complexes are composed of a metal center surrounded by coordinated ligands, forming defined architectures whose shape depends on factors such as oxidation state and the type of ligands. Metal complexes can thus act as globular scaffolds with specific three-dimensional shapes for biomolecular interaction^3^. Moreover, metal complexes can exhibit unique and diverse molecular motifs that are inaccessible to traditional organic small molecules. Finally, their thermodynamic and kinetic parameters can often be optimized to suit a desired therapeutic purpose by adjustment of the auxiliary ligands^4^.

Transition metal compounds, particularly those based on platinum or ruthenium, have been widely investigated as anti-cancer agents^5^. For example, Meggers and co-workers have reported kinetically-inert ruthenium(II) complexes as potent and selective inhibitors of enzyme activity^3^. However, osmium, as the heavier congener of ruthenium, has received comparatively less attention^6^. Compared to ruthenium, osmium complexes are considered to be relatively inert, which could potentially increase their stability under physiological conditions^7^. Sadler, Keppler and co-workers have shown that osmium complexes offer interesting alternatives to their ruthenium counterparts as anticancer agents^6^,^8^. Meanwhile, Meggers and co-workers have suggested that the ability of osmium complexes to offer comparable activities to their ruthenium congeners was due to their similar structures and mechanisms of action^3^. Recently, Che, Lau and co-workers have reported a nitridoosmium (VI) complex that suppressed tumor growth in a nude mice model^9^. These studies demonstrate the potential application of osmium complexes in chemotherapy.

The hypoxia inducible factor (HIF) plays a key role in regulating the hypoxic response in human and other mammals^10^. In normoxic conditions, HIF-1α is degraded at the post-transcriptional level *via* interaction with the von Hippel–Lindau protein^11^. However, under hypoxic conditions, HIF-1α accumulates and dimerizes with HIF-1β, forming a protein heterodimer that complexes with p300 in the nucleus. The HIF-1α–p300 complex then binds to the hypoxia response element (HRE) to activate the transcription of genes involved in angiogenesis, physiological metabolism, cell proliferation and survival^12^,^13^. Accumulating evidence has highlighted the role of HIF-1α and HIF-regulated gene products in tumorigenesis and metastasis^14^. Hence, the HIF-1α pathway has been considered to be an attractive anti-cancer target^15^,^16^. One strategy to inhibit HIF activity is through the disruption of the HIF-1α–p300 protein-protein interaction (PPI)^17^,^18^. However, to our knowledge, no metal-based HIF-1α–p300 PPI inhibitor has been reported yet in the literature. We report herein the identification of an osmium(II) complex as the first metal-based inhibitor of the HIF-1α–p300 interaction.

## Results and Discussion

### Design and preparation of Osmium-based metal complexes

**Figure 1:**
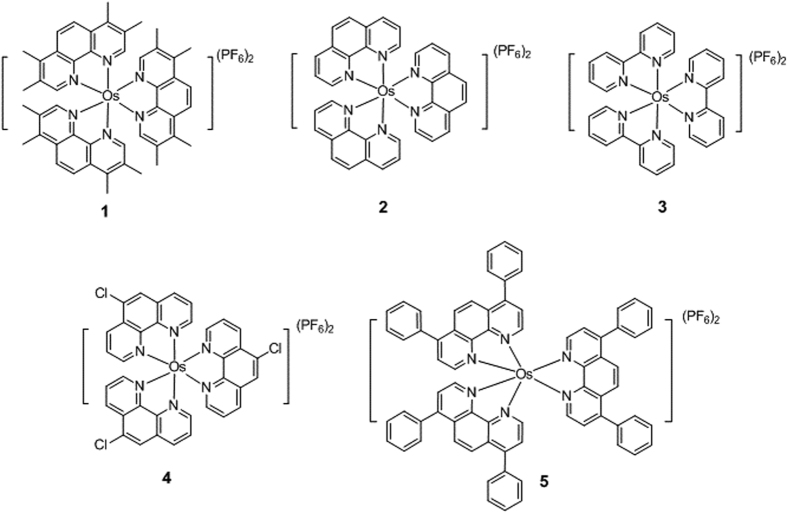
Chemical structures of osmium(II) metal complexes 1–7.

To investigate the ability of the osmium scaffold to act as inhibitors of HIF-1α, osmium(II) complexes with different polypyridyl ligands were designed and synthesized (Fig. 1). Complexes **1**, **2** and **4** all contain three N^N ligands based on 1,10-phenanthroline (phen). Complex **2** carries three unsubstituted phen ligands. Complex **1** bears three 3,4,7,8-tetramethyl-1,10-phenanthroline (phen-*t*Me) ligands, while complex **4** contains three 5-chloro-1,10-phenanthroline (5-Clphen) ligands. Complex **3** carries three smaller 2,2′-bipyridine (bpy) ligands, while complex **5** bears three bulky 4,7-diphenyl-1,10-phenanthroline (4,7-Phphen) ligand. The osmium complexes were prepared by the reaction of sodium hexachloroosmate(IV) hydrate with the corresponding ligands in 1,2-ethanediol under reflux in a nitrogen atmosphere, followed by the addition of NH_4_PF_6_ to precipitate the crude product. Purification by column chromatography on basic alumina using acetonitrile as the eluent afforded the desired products. The complexes were characterized by ^1^H-NMR, ^13^C-NMR, high resolution mass spectrometry (HRMS) and elemental analysis.

### Effects of complexes on the activity of HRE

**Figure 2:**
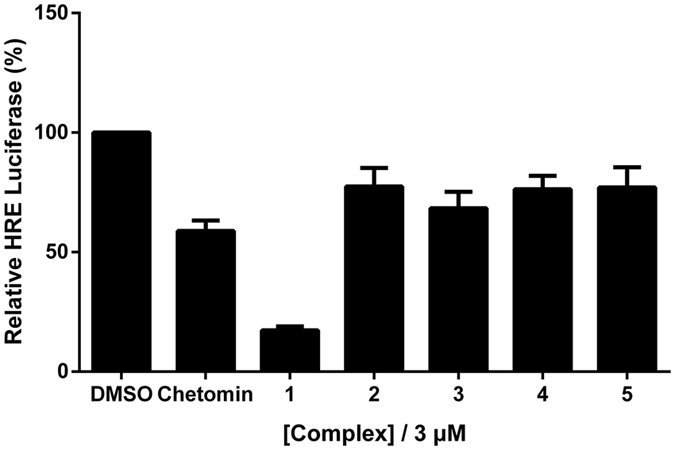
Effect of complexes 1–7 on HRE activity as determined by a dual luciferase reporter assay. Hypoxic MC3T3-E1 cells were treated with 3 μM of complexes or chetomin for 16 h. Error bars represent the standard deviations of results obtained from three independent experiments.

Given that HIF-1α is rapidly degraded under normoxic conditions, we tested the expression of HIF-1α under both normoxic and hypoxic conditions in human embryonic kidney (HEK293T) and mouse osteoblastic (MC3T3-E1) cells. The results showed an increased expression of HIF-1α with time under hypoxia for both cell lines (Supplemental Figs 1 and 2). We then performed the dual luciferase reporter assay to investigate gene transcription in MC3T3-E1 cells. Cells were transiently transfected with pRL-TK and the HIF-1α-directed HRE-luciferase reporter plasmids, and then treated with metal complexes (3 μM) under hypoxic conditions (1% O_2_, 94% N_2_, and 5% CO_2_) for 16 h before measurement. The results showed that the osmium(II) complex [Os(phen-*t*Me)_3_](PF_6_)_2_ **1** exhibited the greatest inhibition of HIF-1α activity, with 73.6% reduction in HRE-driven luciferase activity at 3 μM (Fig. 2). Complexes **3, 6** and **7** showed moderate inhibitory activity in this assay, while complexes **2, 4** and **5** were nearly inactive. Furthermore, complex **1** showed higher potency compared to chetomin, a known HIF-1α–p300 interaction inhibitor^19^.

**Figure 3:**
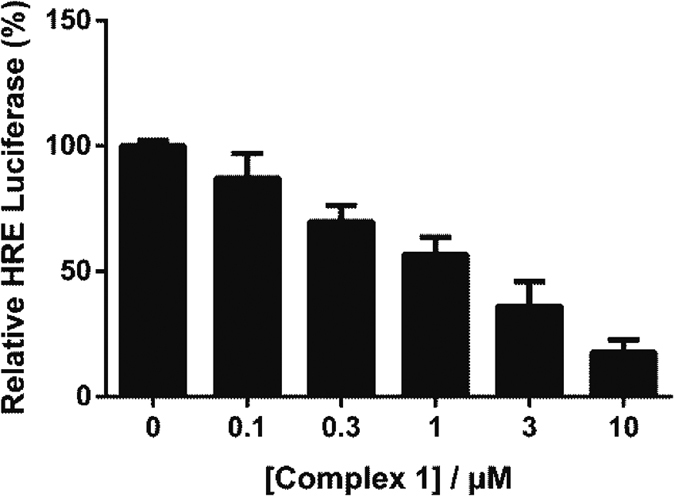
Dose-dependent effect of complex 1 on HRE activity as determined by a dual luciferase reporter assay. Hypoxic MC3T3-E1 cells were treated with indicated concentrations of complex **1** for 16 h. IC_50_ value of complex **1** is *ca.* 1.22 μM. Error bars represent the standard deviations of results obtained from three independent experiments.

The potency of complexes at inhibiting HRE activity in MC3T3-E1 cells was further evaluated using dose-response assays under hypoxia. The results indicated that complex **1** exhibited a dose-dependent inhibition of HRE-driven luciferase activity, with an IC_50_ value of 1.22 μM (Fig. 3), while the IC_50_ value of chetomin against HRE-drive luciferase activity was determined to be 9.83 μM (Supplemental Fig. 3a). Additionally, complexes **2–7** showed IC_50_ values of over 10 μM, indicating that they were less potent than complex **1** (Supplemental Fig. 3b–g). Additionally, the isolated phen-*t*Me ligand of complex **1** barely had any effect on HRE activity (Supplemental Fig. 3h), which demonstrates the role of Os centre in coordinating the bioactive structure of complex **1**. Taken together, these results suggest that complex **1** inhibits HIF-1α activity *in cellulo* with stronger potency. The stability of complex **1** was investigated by ^1^H NMR experiments (Supplemental Fig. 4) and UV-Vis (Supplemental Fig. 5), which revealed that the complex was stable under the testing conditions at least 7 days.

### Structure-activity relationship analysis

Based on the luciferase assay results, a preliminary structure-activity relationship analysis could be performed. Complex **1**, containing three phen*-t*Me ligands, was significantly more active than complex **2** with unsubstituted phen ligands, indicating that the methyl substituents of **1** are important for HIF-1α inhibitory activity. Moreover, 5-chloro (as in **4**) substitution on the phen scaffold resulted in only a slightly improvement in activity compared to **2**. However, increasing the size of N^N ligand to the bulky 4,7-Phphen (as in **5**) ligand reduced in a dramatic decrease in potency. This suggests that excessively bulky functional groups on the N^N ligands may detract from biological activity. Complex **3** bearing the smaller bpy ligand also showed reduced potency compared to **1**, although it was still more active than complex **2**. Finally, complexes **6** and **7**, bearing the extended 11-chloro dipyrido[3,2-*a*:2′,3′-*c*]phenazine and dipyrido[3,2-*a*:2′,3′-*c*]phenazine N^N ligands, respectively, showed minimal activity, suggesting that those N^N ligands are undesirable for potency. Taken together, the results indicate that the HIF-1α inhibitory activity of complex **1** may be attributed its appropriate size and shape that allows it to interact most effectively with its biological target. This is supported by molecular modeling analysis, which suggested that complex **1** binds to the HIF-1α–p300 interface mainly *via* hydrophobic and shape-driven interactions (Supplemental Fig. 6).

The observation that complex **1** displays vastly higher activity compared to complex **2**, despite **1** differing only by the presence of 12 extra methyl groups, is not without literature precedent. For example, Sadler and co-workers have reported very different anticancer activities of structurally similar iminopyridine and azopyridine osmium(II) arene complexes targeting glutathione^20^. Additionally, Meggers and co-workers investigated organoruthenium complexes as inhibitors of the human repair enzyme 8-oxodGTPase (MTH1)^21^. In that study, the introduction of only a single methyl group resulted in a six-fold improvement of potency towards MTH1. Similar observations were revealed in our recent studies on the development of rhodium(III) and iridium(III) complexes as inhibitors of STAT3 and BRD4, respectively^22^,^23^.

### Complex 1 inhibits the interaction of HIF-1α–p300 *in cellulo*

**Figure 4:**
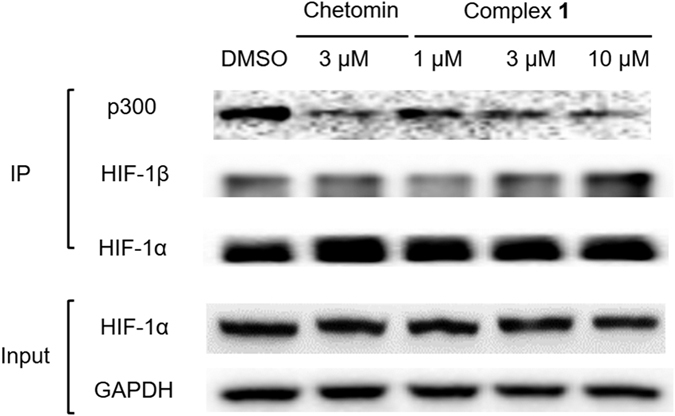
Complex 1 inhibits the interaction of HIF-1α and p300 in MC3T3-E1 cells. Hypoxic MC3T3-E1 cells were treated with the indicated concentrations of complex **1** or chetomin (3 μM) for 16 h. Protein lysates were incubated with anti-HIF-1α magnetic beads, and precipitated proteins were analysed by Western blotting with anti-HIF-1α, anti-p300 or anti-HIF-1β antibodies.

To understand the mechanism of action of complex **1**, we carried out a co-immunoprecipitation experiment. Hypoxic MC3T3-E1 cells were treated with the indicated concentrations of complex **1** or chetomin (3 μM) for 16 h. Cells were lysed and protein lysates were incubated with anti-HIF-1α magnetic beads for 12 h. Precipitated proteins were subjected to SDS-PAGE and analyzed by Western blotting with anti-p300 antibodies, anti-HIF-1α antibodies or anti-HIF-1β antibodies. The results showed that complex **1** interfered with the HIF-1α–p300 interaction as revealed by a dose-dependent reduction of p300 co-precipitated with HIF-1α as the concentration of complex **1** was increased (Fig. 4), with an IC_50_ value of *ca.* 0.41 μM as obtained from densitometry analysis (Supplemental Fig. 7). Furthermore, complex **1** had no appreciable effect on HIF-1α–HIF-1β heterodimer formation (Fig. 4). This result indicates that complex **1** has the ability to block the PPI between HIF-1α and p300 in cancer cells.

### Effect of complex 1 against cell proliferation

In cytotoxicity evaluation, we first evaluated the cytotoxicity of 0.1% (*v*/*v*) DMSO or neat water towards MT3T3-E1 cells under both normoxia and hypoxia. The result showed that both neat water and the indicated concentrations of DMSO had no observable effect on the growth of MT3T3-E1 cells after incubation for 48 h whether under normoxic or hypoxia (Supplemental Fig. 8). Subsequently, the MTT results indicated that complex **1** was highly potent against MC3T3-E1 cells under both normoxic and hypoxic conditions, with IC_50_ values of 10.2 μM and 4.2 μM, respectively, and was moderately cytotoxic towards human liver cancer HepG2 cells and human kidney cancer A498 cells (Supplemental Fig. 9). The relative cell viability decreased significantly after 16 h under hypoxia in treated MC3T3-E1 cells, as compared with cell viability in normoxic conditions, indicating that the toxicity of **1** could be attributed, at least in part, to the inhibition of the HIF-1α activity. We also investigated the effect of complexes on the ROS production as previously described^24^. Flow cytometry result suggested that complex **1** can upregulate the level of ROS (Supplemental Fig. 10), which may also partly contribute to its cytotoxicity on MC3T3-E1 cells.

### Effect of complex 1 on HIF-1α upstream pathways

**Figure 5:**
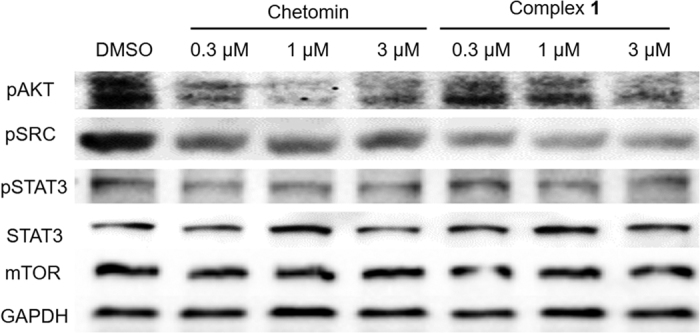
Effect of complex 1 on the phosphorylation and expression of various proteins in MC3T3-E1 cells. Hypoxic MC3T3-E1 cells were treated with the indicated concentrations of complex **1** or chetomin (3 μM) for 16 h. Protein lysates were analyzed by Western blotting with the indicated antibodies.

We performed Western blotting assays to determine whether complex **1** inhibits upstream regulators of the HIF-1α pathway. MC3T3-E1 cells were treated with metal complexes (3 μM) under hypoxic conditions (1% O_2_, 94% N_2_, and 5% CO_2_) for 16 h before measurement. The results showed that complex **1** repressed the phosphorylation of SRC, AKT and STAT3 (Fig. 5), which have been reported to be associated with the expression of HIF-1α^25^,^26^,^27^. Moreover, a dual luciferase assay performed with MC3T3-E1 cells co-transfected with pNF-κB-luc and pRL-TK showed that complex **1** had no discernible effect on the activity of NF-κB (Supplemental Fig. 11). Thus, the results suggest that complex **1** inhibits HIF-1α activity both by interrupting HIF-1α–p300 interaction as well as decreasing HIF-1α gene expression through suppression of SRC, AKT and STAT3 phosphorylation.

## Discussion

PPIs have been regarded as challenging targets for small molecules due to their lack of similarity with conventional ligand-molecule binding sites^28^. In this context, our group has previously developed Group 9 metal-based inhibitors of the PPIs of TNF-α and BRD4^22^,^29^. Therefore, in this work we set out to investigate whether metal complexes could be used as inhibitors of the HIF-1α–p300 PPI, for which no metal-based inhibitor has yet been reported in the literature. To achieve this, we screened five structurally diverse osmium(II) polypyridyl complexes against HIF-1α to see whether or not these scaffolds could potentially bind to the “hot spots” of the HIF-1α–p300 PPI and disrupt HIF-1α expression^18^.

We first tested the complexes against HIF-1α-directed HRE-luciferase expression in MC3T3-E1 cells. Under hypoxic conditions, the osmium(II) complex [Os(phen-*t*Me)_3_](PF_6_)_2_ **1** exhibited the greatest inhibition of HIF-1α activity, with an IC_50_ value of 1.22 μM and with significantly stronger potency to the known HIF-1α–p300 inhibitor, chemotin (IC_50_ = 9.83 μM). Molecular docking analysis of complex **1** at the HIF-1α–p300 interface revealed the absence of salt bridge or hydrogen bonding interactions. This is consistent with the general principle that ligand binding sites. Therefore, we expect that the high degree of shape complementarity between complex **1** and the HIF-1α binding site of p300 is responsible for the ability of the compound to block this PPI and inhibit HIF-1α activity. This is consistent with our SAR analysis, which indicated that complexes bearing bulky or extended N^N ligands (e.g. complexes **5–7**) show limited biological activity, presumably due to their inability to bind within the binding site of p300.

The ability of complex **1** to target the HIF-1α–p300 PPI was verified by conducting a co-immunoprecipitation experiment in hypoxic MC3T3-E1 cells. The experiment showed that complex **1** blocked HIF-1α–p300 interaction *in cellulo*, with an IC_50_ value of ca. 0.41 μM. In contrast, complex **1** had no significant effect on the PPI of the HIF-1α–HIF-1β heterodimer. This suggests that the inhibition of the HIF-1α–p300 PPI by complex **1** involves some degree of specificity and is not simply an artifact arising from non-specific hydrophobic interactions. Further cellular experiments revealed that complex **1** could possibly suppress HIF-1α activity also *via* inhibition of SRC, AKT and STAT3 phosphorylation, which are upstream pathways of HIF-1α. Finally, the higher degree of cytoxicity against hypoxic cells compared to normoxic cells provides further evidence that complex **1** targets HIF-1α, and also raises the possibility that the compound could be developed into a possible anticancer lead, due to hypoxic environment that normally develops around tumor tissues.

In summary, we have synthesized and identified the osmium(II) complex **1** as an inhibitor of the HIF-1α–p300 PPI. Complex **1** inhibited HRE-dependent expression with an IC_50_ of approximately 1.22 μM in hypoxic MC3T3-E1 cells, and was more potent under the same conditions than chetomin, a known HIF-1α–p300 interaction inhibitor. Moreover, complex **1** disrupted the interaction of HIF-1α and p300 *in cellulo* as revealed by a co-immunoprecipitation experiment, and inhibited HIF-1α expression in cells. To our knowledge, complex **1** represents the first metal-based inhibitor of the HIF-1α–p300 PPI. We anticipate that complex **1** could be utilized as a promising scaffold for the further development of more potent HIF-1α inhibitors for anti-cancer treatment.

## Methods

### Materials

High-resolution mass spectrometry was carried out at the Mass Spectroscopy Unit at Hong Kong Baptist University, Hong Kong (China). ^1^H and ^13^C NMR were recorded on a 400 MHz (^1^H) and 100 MHz (^13^C) Bruker instrument using acetonitrile-*d*_3_ or DMSO-*d*_6_ as the solvent. ^1^H and ^13^C chemical shifts were referenced internally to solvent shift (Acetonitrile-*d*_3_:^1^H, δ 1.94, ^13^C, δ 118.7; DMSO-*δ*_6._^1^H, δ 2.50, ^13^C, δ 39.5) Coupling constants are typically ± 0.1 Hz for ^1^H-^1^H and ± 0.5 Hz for ^1^H-^13^C couplings. The following abbreviations are used for convenience in reporting the multiplicity of NMR resonances: s, singlet; d, doublet; t, triplet; q, quartet; m, multiplet. All NMR data was acquired and processed using standard Bruker software (Topspin). All the complexes were immersed in dimethyl sulfoxide (DMSO), and the concentration of DMSO in all bioassays was 0.1% (*v*/*v*). The elemental analysis test of complexes was performed in Atlantic Microlab, Inc. (USA).

### The general preparation of [Os(N^N)_3_](PF_6_)_2_

In a typical preparation, Na_2_OsCl_6_ (45 mg, 0.10 mmol) and the corresponding N^N (0.33 mmol, 3.3 equiv) ligand were suspended in dry 1,2-ethanediol (5 mL) in flask equipped with a magnetic stir bar. The mixture was heated to reflux and stirred for 4 h under a N_2_ atmosphere. Following the reaction, the mixture was cooled to room temperature, saturated aqueous NH_4_PF_6_ (10 mL) was added to precipitate the product as the hexafluorophosphate salt. The precipitate was washed with water and diethyl ether to give the crude product, which was purified by column chromatography on basic alumina using acetonitrile as the eluent.

### Preparation of the ligand of complexes 6 and 7

A mixture of 1,10-phenanthroline-5,6-dione (5.00 mmol, 1 eq) and 4-chlorobenzene-1,2-diamine or benzene-1,2-diamine (6.00 mmol, 1.2 eq) in ethanol were heated to reflux overnight. The reaction mixture was slowly cooled down to room temperature, and the solid that precipitated from solution was filtered off, washed with cold ethanol, and suction-dried to obtain the desired product that was directly used in the next step.

### The general preparation of [Os(bpy)_2_Cl_2_]_2_

In a typical preparation^30^, the (NH_4_)_2_OsCl_6_ salt (1 mmol) was dissolved in 20 mL of ethylene glycol with 2 molar equiv of bpy (2 mmol). The solution was refluxed under argon for 45 min. After cooling to room temperature, 10 mL of aqueous sodium dithionite (2.0 mol) was added to the reaction mixture to reduce Os(III) to Os(II). The precipitate formed was isolated by filtration, washed with water and a large amount of diethyl ether, and dried.

### The general preparation of [Os(bpy)_2_(N^N)](PF_6_)_2_

In a typical preparation^30^, the dimer [Os(bpy)_2_Cl_2_]_2_ (0.20 mmol, 1 eq) the corresponding N^N (0.44 mmol, 2.2 equiv) ligand was dissolved in 9 mL of ethylene glycol in flask equipped with a magnetic stir bar. The solution was refluxed at 180 °C under nitrogen for 5 h and then cooled to room temperature. The mixture was added to excess NH_4_PF_6_ to precipitate the product as the hexafluorophosphate salt. The precipitate was washed with water and diethyl ether to give the crude product, which was purified by column chromatography on basic alumina using acetonitrile as the eluent. The purity (≥95%) of complex **2**–**5** was determined by Agilent 1200 high-performance liquid chromatography (HPLC) system using an Agilent C18 column (4.6 mm × 250 mm, 5 μm).

### Complex 1: [Os(phen-tMe)_3_](PF_6_)_2_

Yield: 58.9%. ^1^H NMR (400 MHz, acetonitrile-*d*_3_) *δ* 8.34 (s, 6H), 7.57 (s, 6H), 2.84 (s, 18H), 2.19 (s, 18H); ^13^C NMR (100 MHz, acetonitrile) 151.4, 148.4, 143.5, 134.3, 129.3, 124.0, 117.0, 16.4, 13.4; MALDI-TOF-HRMS: Calcd. for C_48_H_48_N_6_OsPF_6_ [M – PF_6_]^+^: 1045.3194; found: 1045.3115. Anal.: (C_48_H_48_N_6_OsP_2_F_12_ + 0.5H_2_O) C, H, N: calcd. 48.12, 4.12, 7.01; found 48.16, 4.21, 7.00.

### Complex 2: [Os(phen)_3_](PF_6_)_2_

Reported^31^. HPLC purity ≥95% (Supplemental Fig. 12a). Yield: 58%. ^1^H NMR (400 MHz, acetonitrile-*d*_3_) *δ* 8.41 (d, *J* = 8.4 Hz, 6H), 8.28 (s, 6H), 7.59 (d, *J* = 8.0 Hz, 6H), 7.59 (t, *J* = 6.8 Hz, 6H); ^13^C NMR (101 MHz, acetonitrile) *δ* 152.07, 149.75, 136.20, 130.94, 127.91, 117.01; MALDI-TOF-HRMS: Calcd. for C_36_H_24_F_6_N_6_OsP [M – PF_6_]^+^: 877.1319; found: 877.1212.

### Complex 3: [Os(bpy)_3_](PF_6_)_2_

Reported^32^. HPLC purity ≥95% (Supplemental Fig. 12b). Yield: 46%. ^1^H NMR (400 MHz, acetonitrile-*d*_3_) *δ* 8.39 (d, *J* = 8.0 Hz, 3H), 7.78 (t, *J* = 8.0 Hz, 3H), 7.55 (d, *J* = 6.0 Hz, 3H), 7.22 (t, *J* = 6.0 Hz, 3H); ^13^C NMR (101 MHz, acetonitrile) *δ* 160.27, 152.17, 138.48, 129.39, 125.82, 118.70. MALDI-TOF-HRMS: Calcd. for C_30_H_24_F_6_N_6_OsP [M – PF_6_]^+^: 805.1319; found: 805.1212.

### Complex 4: [Os(phen-Cl)_3_](PF_6_)_2_

Reported^33^. HPLC purity ≥95% (Supplemental Fig. 12c). Yield: 62%. ^1^H NMR (400 MHz, acetonitrile-*d*_3_) *δ* 8.62 (d, *J* = 7.8 Hz, 3H), 8.56−8.45 (m, 3H), 8.34 (d, *J* = 8.0 Hz, 3H), 8.01−8.00 (m, 3H), 7.96−7.93 (m, 3H), 7.67−7.65 (m, 3H), 7.59−7.56 (m, 3H); ^13^C NMR (101 MHz, acetonitrile) *δ* 154.92, 154.86, 154.84, 154.79, 154.34, 154.21, 152.16, 150.76, 137.31, 135.10, 133.26, 131.91, 131.88, 131.07, 131.04, 128.50, 128.49, 128.13, 128.09, 128.07, 128.03; MALDI-TOF-HRMS: Calcd. for C_36_H_21_Cl_3_F_6_N_6_OsP [M – PF_6_]^+^: 979.0150; found: 979.0310.

### Complex 5: [Os(4,7-Phphen)_3_](PF_6_)_2_

Reported^34^. HPLC purity ≥95% (Supplemental Fig. 12d). Yield: 48%. ^1^H NMR (400 MHz, acetonitrile-*d*_3_) *δ* 8.23 (s, 6H), 8.19 (d, J = 5.6 Hz, 6H), 7.68−7.56 (m, 36H). ^13^C NMR (101 MHz, Acetonitrile) δ 152.82, 151.60, 149.87, 136.41, 131.05, 130.66, 130.20, 130.10, 127.25, 127.16. MALDI-TOF-HRMS: Calcd. for C_72_H_48_F_6_N_6_OsP [M – PF_6_]^+^: 1333.3197; found: 1333.3191. Anal.: (C_72_H_48_F_12_N_6_OsP_2_) C, H, N: calcd. 57.83, 3.37, 5.62; found 57.72, 3.19, 5.73.

### Complex 6: [Os(bpy)_2_(dppz-Cl)](PF_6_)_2_

Reported^35^. Yield: 60% ^1^H NMR (400 MHz, acetonitrile-*d*_3_) *δ* 9.45−9.42 (m, 2H), 8.85 (d, *J* = 8.4 Hz, 2H), 8.81 (d, *J* = 8.0 Hz, 2H), 8.50−8.46 (m, 3H), 8.42 (d, *J* = 2.0 Hz, 1H), 8.13−8.05 (m, 5H), 8.03−7.96 (m, 6H), 7.58−7.55 (m, 2H), 7.36−7.31 (m, 2H). ^13^C NMR (100 MHz, acetonitrile-*d*_3_) 160.2, 160.1, 154.4, 154.3, 154.2, 154.1, 152.4, 152.1, 143.5, 142.1, 141.9, 141.4, 138.7, 138.6, 138.5, 134.1, 134.0, 133.9, 132.3, 131.8, 131.7, 129.3, 129.2, 129.1, 129.0, 122.6, 125.6. MALDI-TOF-HRMS: Calcd. for C_38_H_25_N_8_ClOsPF_6_ [M – PF_6_]^+^: 965.1147; found: 965.0513; Anal.: (C_38_H_25_N_8_ClOsP_2_F_12_ + H_2_O) C, H, N: calcd. 40.49, 2.41, 9.94; found 40.40, 2.65, 9.86.

### Complex 7: [Os(bpy)_2_(dppz)](PF_6_)_2_

Reported^36^. Yield: 59%. ^1^H NMR (400 MHz, acetonitrile-*d*_3_) *δ* 9.44 (dd, *J* = 0.8 Hz, 8.0 Hz, 2H), 8.55−8.47 (m, 6H), 8.16−8.13 (m, 2H), 8.10 (dd, *J* = 1.2 Hz, 5.6 Hz, 2H), 7.95−7.91 (m, 2H), 7.86−7.76 (m, 6H), 7.63 (d, *J* = 5.6 Hz, 2H), 7.41−7.39 (m, 2H), 7.19−7.15 (m, 2H); ^13^C NMR (100 MHz, acetonitrile) *δ* 158.7, 158.5, 152.5, 152.4, 151.0, 150.8, 142.4, 139.8, 137.0, 136.9, 132.5, 132.2, 130.8, 129.3, 127.7, 127.6, 127.4, 124.2, 124.1. MALDI-TOF-HRMS: Calcd. for C_38_H_26_N_8_Os [M – 2PF_6_]^+^: 786.1891; found: 786.1838; Anal.: (C_38_H_26_N_8_OsP_2_F_12_ + 3.5H_2_O) C, H, N: calcd. 40.11, 2.92, 9.85; found 40.1, 2.63, 9.77.

### Stability analysis of complex 1

Complex **1** was incubated in DMSO-*d*_6_/D_2_O (*v*/*v* = 9:1) at 298 K for seven days, and ^1^H NMR spectra were recorded daily. ^1^H NMR experiments were carried out on a 400 MHz (^1^H) Bruker instrument. Alternatively, complex **1** was incubated in acetonitrile/Tris-HCl buffer (*v*/*v* = 8:2, 20 μM) 298 K for seven days and absorption spectra were recorded on a Cary UV-100 Spectrophotometer daily.

### Cell culture

Cells were cultivated in DMEM medium or RPMI-1640 medium with 1% penicillin (100 units/mL)/streptomycin (100 μg/mL) containing 10% fetal bovine serum (FBS). Cells were maintained at a cell density of 4–5 × 10^5 ^cells/mL. Cells were cultured in an atmosphere of 5% CO_2_ at 37 °C. To induce hypoxia, cells were placed in a hypoxia chamber (Whitley VA500 Workstation, Don Whitley Scientific Ltd., UK) with an atmosphere of 1% O_2_, 94% N_2_, and 5% CO_2_.

### Theoretical calculations

The molecular structure of **1** was optimized at the density functional theory (DFT) level using the BP86 functional. The def2-SVP basis sets were used for the H, C, and N atoms, while the def2-TZVP(-f) basis sets were used for the Os atom^37^. The resolution of the identity algorithm was used to accelerate the DFT calculation^38^. Zero-order regular approximation (ZORA) was employed to account for relativistic effects. Tight SCF convergence (10^−8^ au) was used for all calculations. All the calculations were performed using the ORCA software package (version 3.0.3)^39^.

### Purity experiment

The purity of complexes 2–**5** was determined to be ≥95% by use of an Agilent 1200 high-performance liquid chromatography (HPLC) system using an Agilent C18 column (4.6 mm × 250 mm, 5 μm). Mobile phase A was Milli-Q water (with 0.1% *v*/*v* trifluoroacetic acid (TFA)) and mobile phase B was acetonitrile (with 0.1% *v*/*v* TFA), respectively. The flow rate was 1.0 mL/min and UV absorbance was monitored at 280 nm.

### Molecular modeling

Molecular docking was performed by using the ICM-Pro 3.6-1d program (Molsoft)^40^,^41^. According to the ICM method, the molecular system was described by using internal coordinates as variables. Energy calculations were based on the ECEPP/3 force field with a distance-dependent dielectric constant. The biased probability Monte Carlo (BPMC) minimization procedure was used for global energy optimization. The BPMC global-energy-optimization method consists of (1) a random conformation change of the free variables according to a predefined continuous probability distribution; (2) local-energy minimization of analytical differentiable terms; (3) calculation of the complete energy including nondifferentiable terms such as entropy and solvation energy; (4) acceptance or rejection of the total energy based on the Metropolis criterion and return to step (1).

### Transient transfection

MC3T3-E1 cells were seeded in six well plates 24 h before transfection. HRE-luciferase plasmid (4 μg) or pNF-κB-luc (4 μg), pRL-TK plasmid (4 μg) and TurboFect reagent (6 μL) were mixed together in serum-free RPMI 1640 medium and the resulting solution was incubated for 20 min at room temperature. The mixture was the added dropwise to the MC3T3-E1 cells in the wells. The cells were incubated for 32 h at 37 °C in a CO_2_ incubator before use.

### Dual luciferase reporter assay

The inhibition of HIF-1α or NF-κB activity was assayed by a reporter assay using a dual luciferase reporter assay system (Promega, Madison, WI, USA), as previously described^42^,^43^. Transiently transfected cells were treated with complex or chetomin in hypoxic conditions for 16 h before measurement. Cells treated with DMSO (0.1%, V/V) were served as a vehicle control. Luciferase activity was integrated over a 7.5 second period and measured using a spectrophotometer (Spectra-max M5, Molecular Devices, USA). The results were standardization with the activity of Renilla luciferase. All data are expressed as means ± SD.

### Intracellular ROS detection

Intracellular ROS production was analyzed by flow cytometry analysis as previously reported^24^. Briefly, MC3T3-E1 cells were seeded at the density of 1 × 10^5^ cells per well in 12-well plates, and then cultured with 0.1% DMSO or 3 μM of complexes (DMSO/medium = 1:1000, *v*/*v*) for 16 h under hypoxia conditions. After treatment, cells were washed two times with PBS and collected, then incubated with 5 μM DCFH-DA at 37 °C for 20 min in the dark and analyzed using the BD Accuri™ C6 Plus flow cytometer within 1 h.

### Western blotting

Cells were exposed to the indicated concentrations of **1** or DMSO overnight. Cells were washed three times with ice-cold PBS, resuspended in RIPA lysis buffer, and incubated on ice for 30 min. Cell debris was removed by centrifugation at 14,000 rpm for 30 min at 4 °C, and the protein concentration of the supernatant was determined with Bio-Rad protein assay dye reagent (Bio-Rad). Protein samples were prepared. Western blotting analysis was performed as described^24^.

### Co-immunoprecipitation assay

The assay was performed as previous described^44^. Briefly, MC3T3-E1 cells were seeded at the density of 1 × 10^6^ cells in a 6 mL culture dish. Cells were treated with the indicated concentrations of complex **1** or 10 μM of chetomin for 16 h under hypoxic conditions (1% O_2_, 94% N_2_, and 5% CO_2_). Cells were lysed and collected the protein samples. The concentration of protein samples was detected using the Pierce BCA protein assay kit. 30 μg of each protein sample were incubated 12 h with 10 μL pre-incubated anti-p300 or anti-HIF-1α magnetic beads according to the manufacturer’s protocol. The complex was washed 5 times to elute non-specific and non-cross-linked antibodies. Then, the precipitated proteins were subjected to SDS-PAGE and analysed by Western blotting with anti-p300 (1:1,000, Santa Cruz), anti-HIF-1α (1:1,000, Santa Cruz) and anti-HIF-1β (1:1,000, Santa Cruz).

### Cytotoxicity experiment

The experiment was performed as previously described^47^. Cells were seeded at the density of 5,000 cells per well in 96-well plates and until they reached 30–50% confluence. Then medium was replaced with fresh medium. Complex **1** dissolved in DMSO was added to cells at final concentrations ranging from 10 nM to 100 μM under normoxic or hypoxic conditions for 48 h. The final concentration of DMSO was 0.1% (*v*/*v*). Added MTT in per well at the final concentration 0.5 mg/mL for a further 4 h. Remove the medium from the cells before adding 100 μL DMSO in per well. Before starting test, shake the plate 10 mins at room temperature in the dark. The cytotoxicity of complex **1** was exhibited as the percentage of absorbance in SpectraMax M5 microplate reader at 490 nm and 600 nm.

## Additional Information

**How to cite this article:** Yang, C. *et al*. Anticancer osmium complex inhibitors of the HIF-1α and p300 protein-protein interaction. *Sci. Rep.* **7**, 42860; doi: 10.1038/srep42860 (2017).

**Publisher's note:** Springer Nature remains neutral with regard to jurisdictional claims in published maps and institutional affiliations.

## Supplementary Material

Supplementary Information
