# Supplementary material for: Anticancer osmium complex inhibitors of the HIF-1α and p300 protein-protein interaction
Source: Sci Rep. 2017 Feb 22;7:42860. doi: 10.1038/srep42860 (PMC5320473; doi:10.1038/srep42860)

**Anticancer osmium complex inhibitors of the HIF-1α and p300 protein-protein interaction**

Chao Yang^1,*^, Wanhe Wang^2,*^, Guo-Dong Li^1^, Hai-Jing Zhong^1^, Zhen-Zhen Dong^2^, Chun-Yuen Wong^3^, Daniel W. J. Kwong^2^, Dik-Lung Ma^2^ & Chung-Hang Leung^1^

^1^State Key Laboratory of Quality Research in Chinese Medicine, Institute of Chinese Medical Sciences, University of Macau, Macao, China

^2^Department of Chemistry, Hong Kong Baptist University, Kowloon Tong, Hong Kong, China

^3^Department of Biology and Chemistry, City University of Hong Kong, Tat Chee Avenue, Kowloon, Hong Kong SAR, China

^*^These authors contributed equally to this work.

Correspondence and requests for materials should be addressed to C.-H.L. (email: duncanleung@umac.mo) or D.-L.M. (email: edmondma@hkbu.edu.hk)

**SUPPLEMENTARY FIGURES**

**
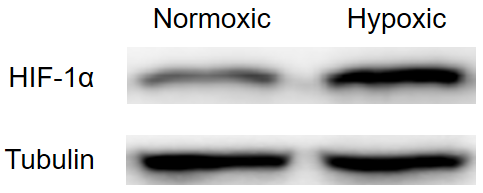
**

**Supplementary Figure 1.** HIF-1α expression under normoxic and hypoxic conditions. Normoxic and hypoxic MC3T3-E1 cell extracts were prepared and protein samples were collected. Western blotting analysis was performed.


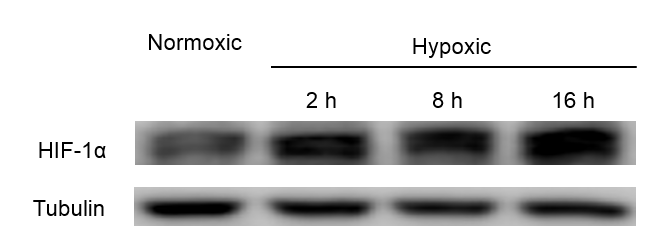


**Supplementary Figure 2.** HIF-1α expression under normoxic and hypoxic conditions. Normoxic and hypoxic HEK293T cell extracts were prepared and protein samples were collected. Western blotting analysis was performed.

**
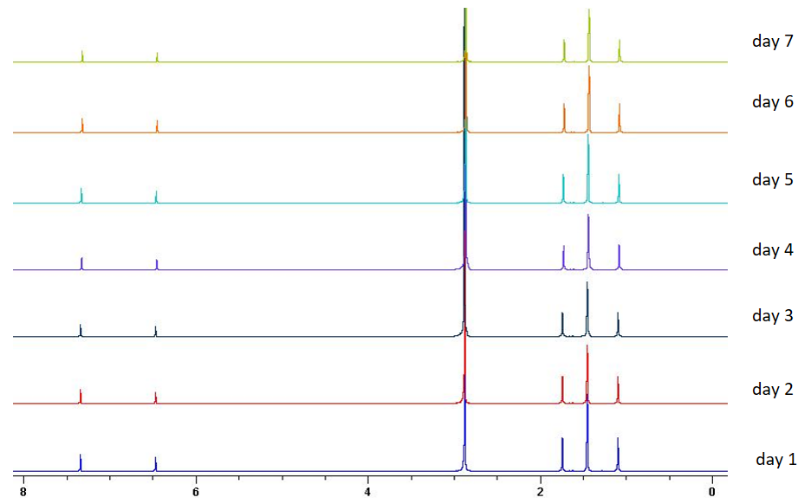
Supplementary**
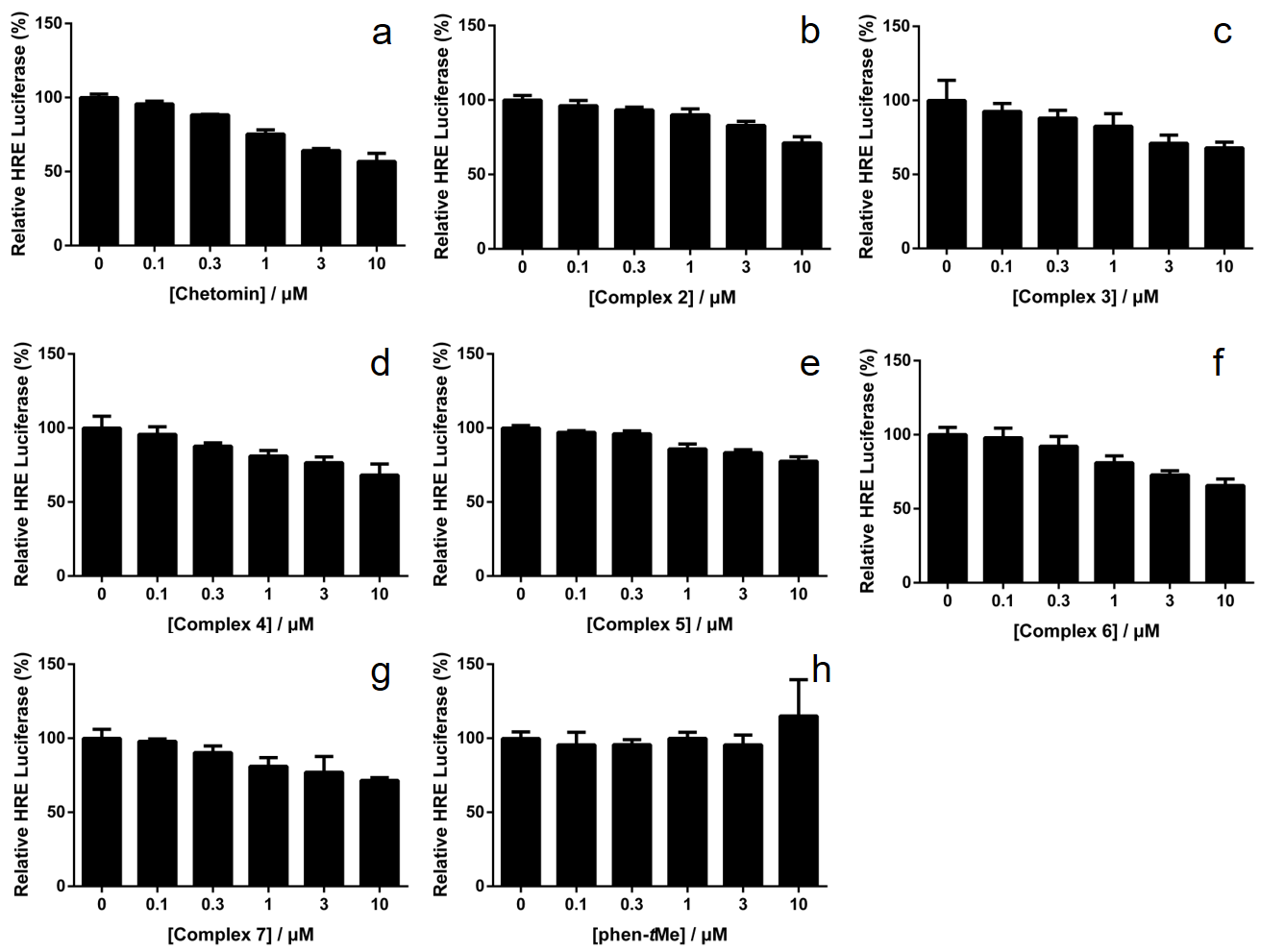
**Figure 3.** Dose-dependent effect of chemotin, complexes **2**–**7** and phen-*t*Me ligand on HRE activity as determined by a dual luciferase reporter assay. Hypoxic MC3T3-E1 cells were treated with indicated concentrations of chemotin (a), complexes **2**–**7** (b-g) and ligand (c) for 16 h. IC_50_ value of chemotin is *ca.* 9.83 µM, IC_50_ values of complexes **2–7** and phen-*t*Me ligand were above 10 μM. Error bars represent the standard deviations of results obtained from three independent experiments.

**Supplementary Figure 4.** ^1^H NMR spectra of complex **1** at a concentration of 5 mM in 90% [*d*_6_]DMSO/10% D_2_O at 298 K over 7 days.

**Supplementary**
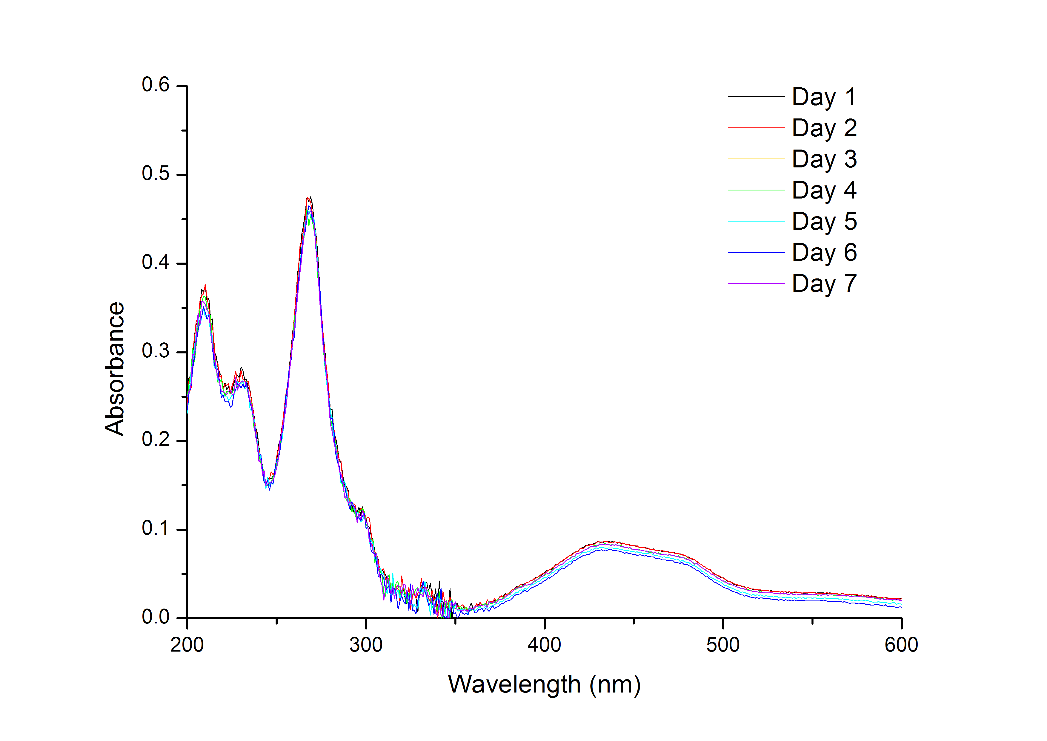
**Figure 5.** UV/Vis absorption of complex **1** at a concentration of 20 μM in 80% acetonitrile/20% Tris-HCl buffer (20 mM containing 20 mM NaCl, pH 7.5) at 298 K over 7 days.

**
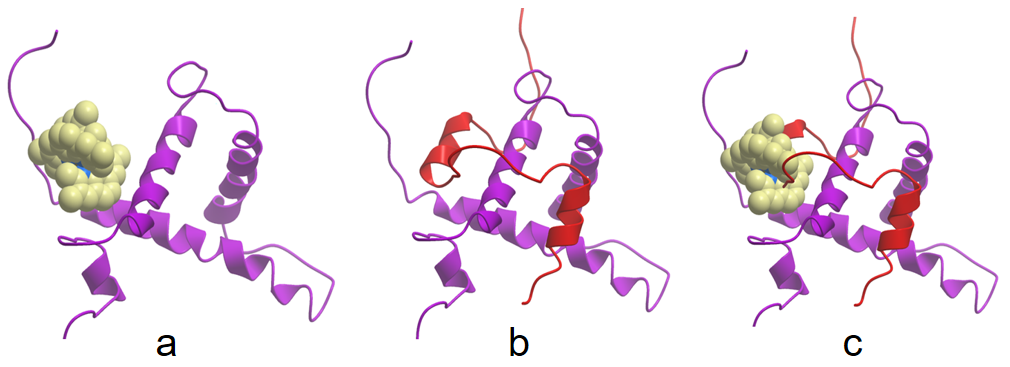
**

**Supplementary Figure 6.** Low-energy binding conformations of (a) p300 and **1**, (b) HIF-1α and p300 and (c) superimposition of (a) and (b) as generated by molecular docking. The protein is depicted as a ribbon representation (red and purple), while **1** is depicted as a space-filling representation showing carbon (beige) and nitrogen (blue).


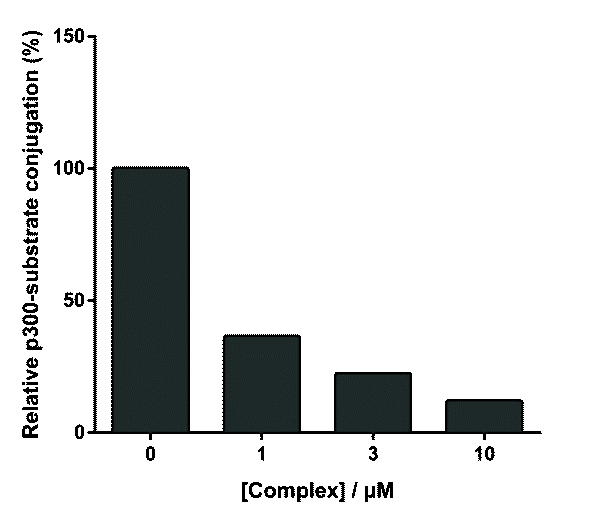


**Supplementary**
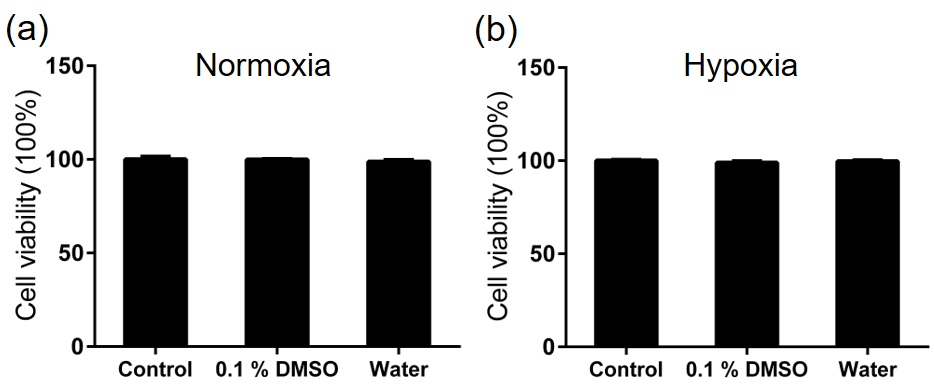
**Figure 7.** Densitometry analysis of the cell-based Western blot showing complex **1**-mediated reduction of HIF-1α-p300. Approximate IC_50_ value = 0.41 μM.

**Supplementary Figure 8.** The cytotoxicity effect of neat water or 0.1% DMSO in water on MC3T3-E1 cells under (a) normoxic and (b) hypoxic conditions as determined by a MTT assay. MC3T3-E1 cells were incubated with neat water or 0.1% DSMO in water for 48 h under normoxic or hypoxic conditions. Control is the fresh DMEM medium with 1% penicillin containing 1% FBS.


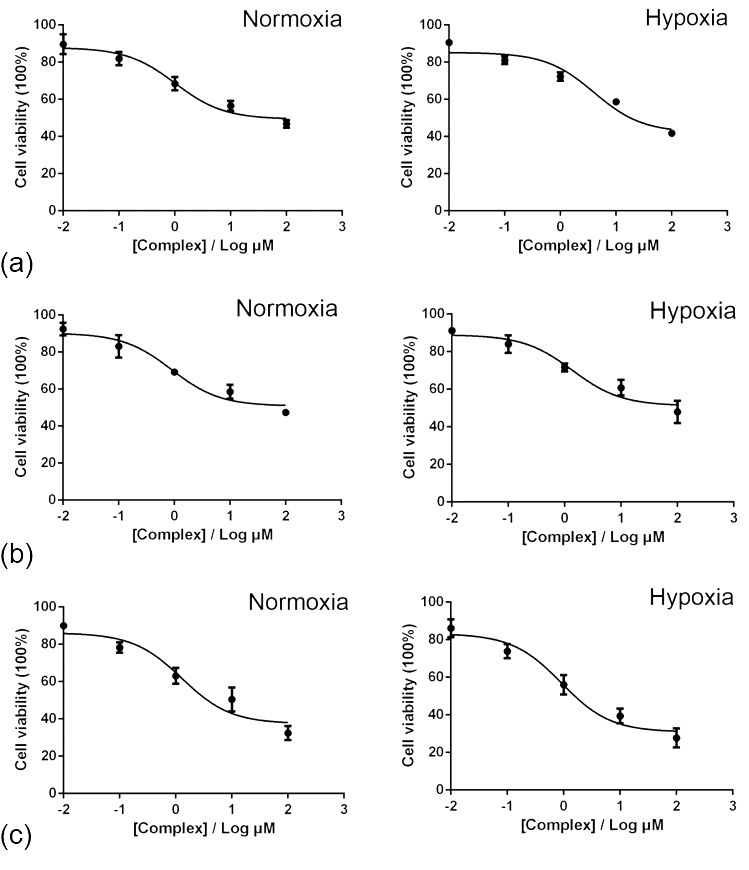


**Supplementary Figure 9.** The cytotoxicity effect of complex **1** on (a) A498 cells, (b) HepG2 cells and (c) MC3T3-E1 cells under normoxic and hypoxic conditions as determined by an MTT assay. Cells were exposed to the indicated concentrations of complex **1** for 48 h. Complex **1** inhibited the growth under normoxic and hypoxia conditions of A498 cells with IC_50_ values of 36.8 μM and 31.1 μM, respectively, HepG2 cells with IC_50_ values of 49.6 μM and 36.2 μM, respectively, and MC3T3-E1 cells with IC_50_ values of 10.2 μM and 4.2 μM, respectively. Error bars represent the standard deviations of the results from three independent experiments.


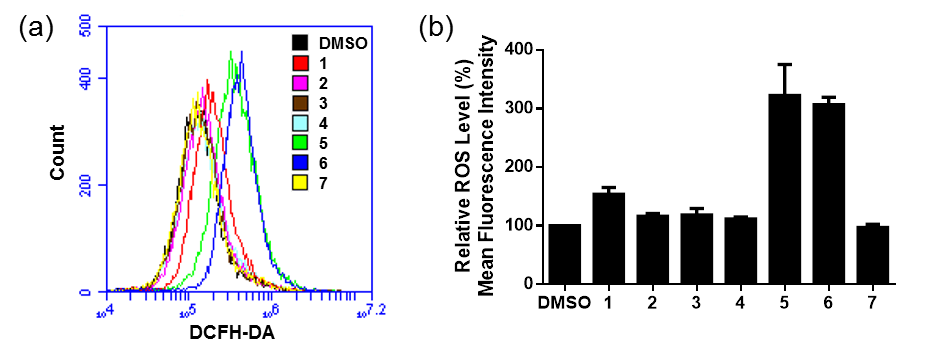


**Supplementary**
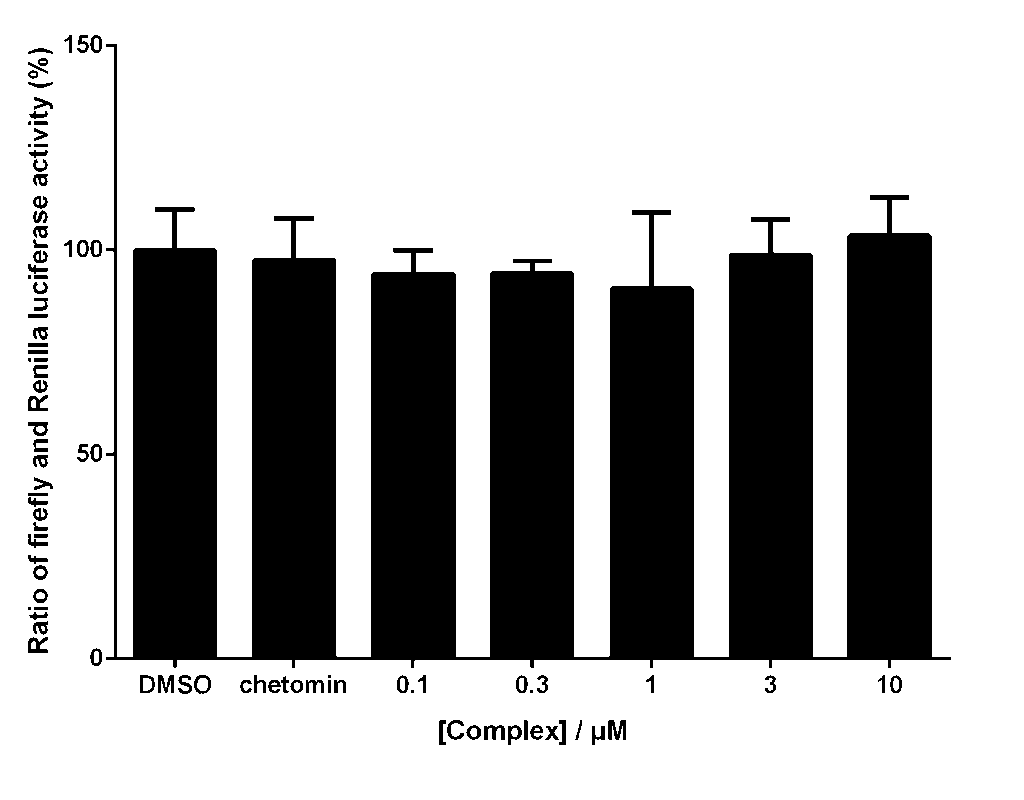
**Figure 10.** The intracellular ROS production were determined by flow cytometry analysis. MC3T3-E1 cells were treated with 0.1% DMSO or 3 µM of complexes for 16 h under hypoxia conditions. Cells were analyzed by flow cytometry upon DCFH-DA staining. (a) Flow cytometric histogram. (b) Quantification of mean fluoresence intensity.

**Supplementary Figure 11.** Effect of complex **1** on NF-κB activity as determined by a dual luciferase reporter assay. Hypoxic MC3T3-E1 cells were treated with indicated concentrations of complex **1** or chemotin (10 μM) stimulation with TNF-α (25 ng/mL) for 16 h. Error bars represent the standard deviations of results obtained from three independent experiments.


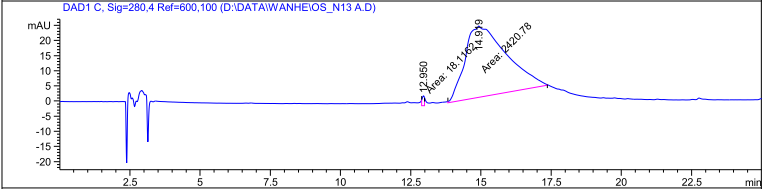


a


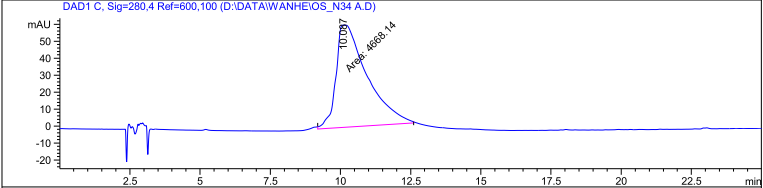


b


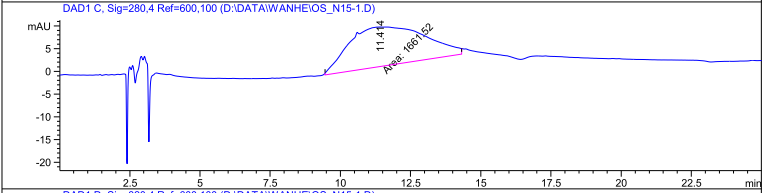

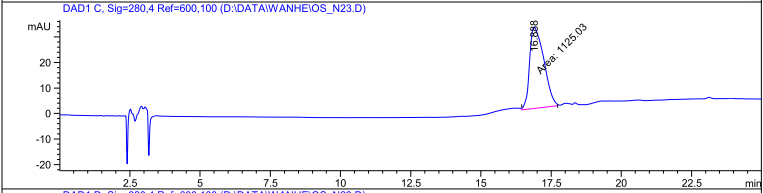


c

d

**Supplementary Figure 12.** HPLC trace for complexes **2**-**5**. (a) complex **2** (10–40% acetonitrile with 0.1% TFA over 10 min, 40–65% acetonitrile with 0.1% TFA from 10 min to 15 min, and 40–65% acetonitrile with 0.1% TFA from 15 min to 25 min); (b) complex **3** (10–40% acetonitrile with 0.1% TFA over 10 min, 40–65% acetonitrile with 0.1% TFA from 10 min to 15 min, and 40–65% acetonitrile with 0.1% TFA from 15 min to 25 min); (c) complex **4** (10–30% acetonitrile over 5 min, 30–90% acetonitrile from 5 min to 15 min, and 90–95% acetonitrile from 15 min to 25 min); (d) complex **5** (10–30% acetonitrile over 5 min, 30–90% acetonitrile from 5 min to 15 min, and 90–95% acetonitrile from 15 min to 25 min).

**Full, uncut gel images for Figures 4 and 5.**


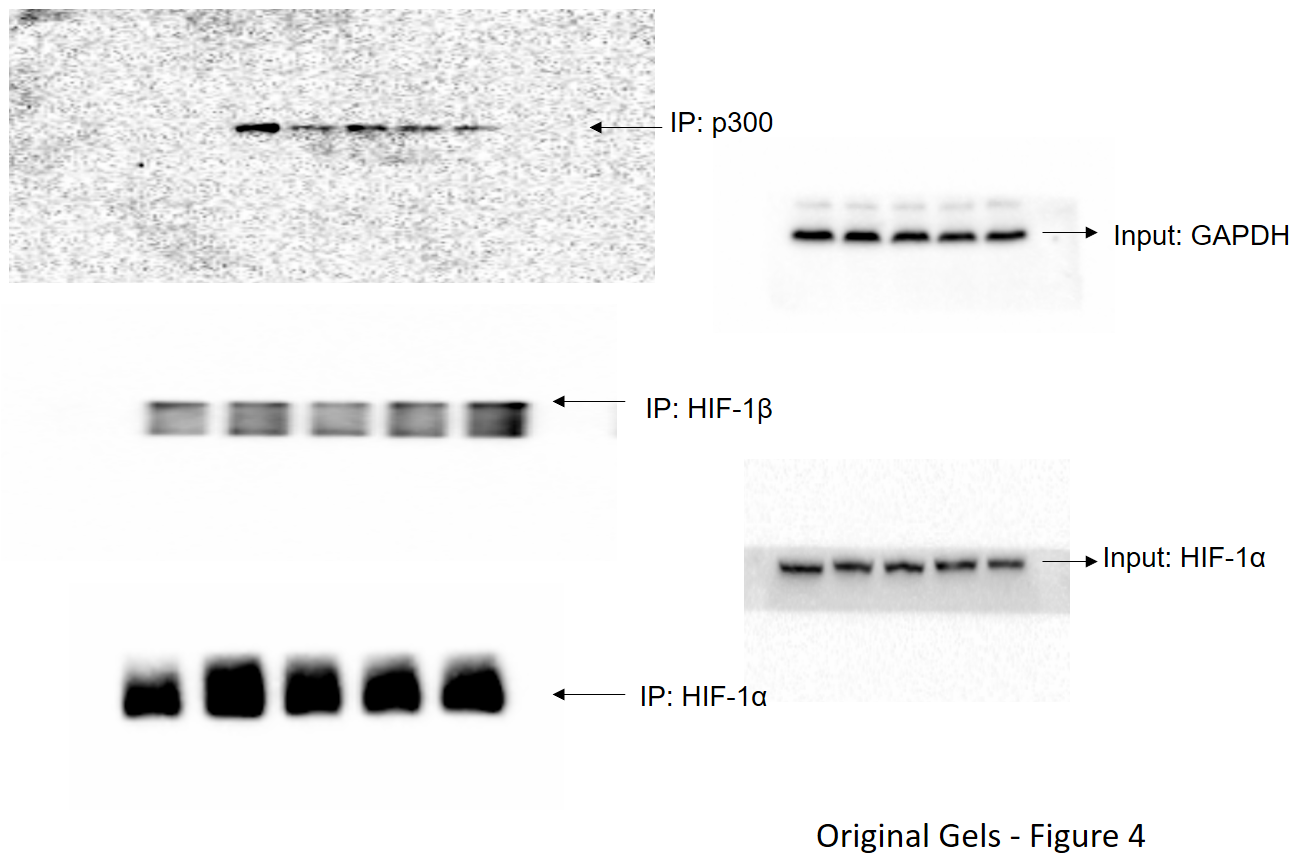


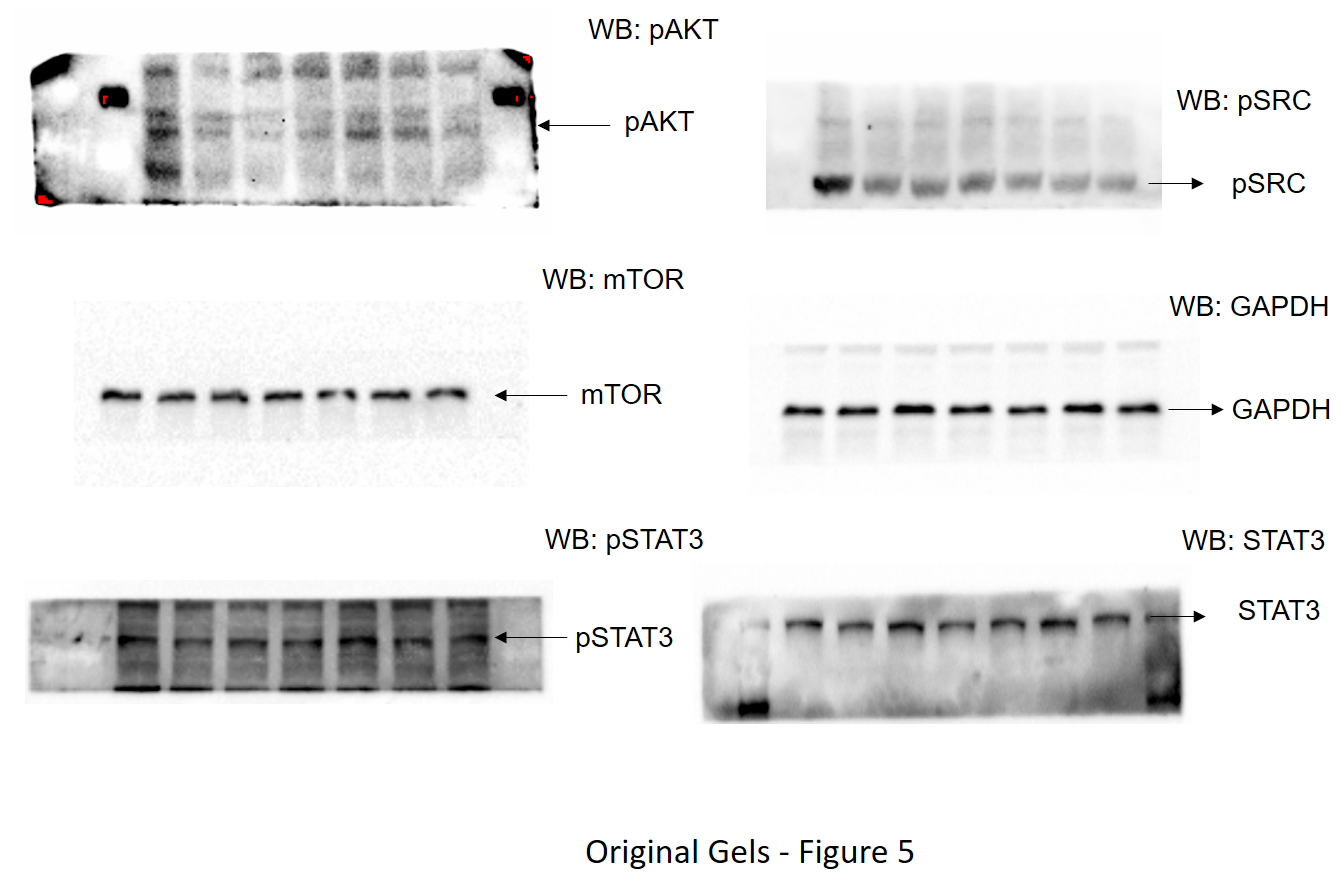

Supplement: Supplementary Information [file srep42860-s1.doc]
